# Pathological Complete Response to Nivolumab Plus Ipilimumab Combination Therapy in Advanced Renal Cell Carcinoma With Inferior Vena Cava Tumor Thrombus

**DOI:** 10.1002/iju5.70200

**Published:** 2026-05-20

**Authors:** Takuto Tsuchiya, Go Kimura, Hayato Takeda, Jun Akatsuka, Hiroya Hasegawa, Yuka Toyama, Takashi Sakatani, Akira Shimizu, Ryuji Ohashi, Yukihiro Kondo

**Affiliations:** ^1^ Department of Urology Nippon Medical School Tokyo Japan; ^2^ Department of Diagnostic Pathology Nippon Medical School Hospital Tokyo Japan; ^3^ Department of Analytic Human Pathology Nippon Medical School Tokyo Japan; ^4^ Department of Integrated Diagnostic Pathology Nippon Medical School Tokyo Japan

**Keywords:** inferior vena cava tumor thrombus, nivolumab plus ipilimumab, pathological complete response, programmed death‐ligand 1 expression, renal cell carcinoma

## Abstract

**Introduction:**

Nivolumab plus ipilimumab is a standard first‐line regimen for advanced renal cell carcinoma; however, primary tumors often respond less favorably to this combination than to immune checkpoint inhibitor–tyrosine kinase inhibitor regimens, and pathological complete response is rare.

**Case Presentation:**

A 78‐year‐old man presented with abdominal pain. Computed tomography revealed a 91‐mm right renal mass with a Neves level 3 inferior vena cava tumor thrombus. Needle biopsy confirmed clear cell renal cell carcinoma with diffuse programmed death‐ligand 1 expression, and both the tumor proportion and combined positive scores were approximately 100%. Nivolumab plus ipilimumab was administered. After 21 months, the primary tumor decreased in size by 68.1% and tumor thrombus regressed from level 3 to 1. Histopathological examination following radical nephrectomy with tumor thrombectomy demonstrated a pathological complete response.

**Conclusion:**

Diffuse programmed death‐ligand 1 expression on pretreatment biopsy may have been associated with a favorable response to nivolumab plus ipilimumab.

## Keynote Message

This case demonstrates that nivolumab plus ipilimumab can induce a pathological complete response in advanced renal cell carcinoma with an inferior vena cava tumor thrombus. Although this regimen is generally considered less effective for primary tumors, diffuse programmed death‐ligand 1 expression in pretreatment biopsy tissue may have been associated with a favorable response in this case. These findings suggest that immunohistochemical features on pretreatment biopsy may provide useful information when considering treatment strategies in selected patients.

**Abbreviations**
CR: complete response
CT: computed tomography
ICI: immune checkpoint inhibitor
IVC: inferior vena cava
IVC‐TT: inferior vena cava tumor thrombus
PD‐L1: programmed death‐ligand 1
RCC: renal cell carcinoma

## 1 Introduction

Renal cell carcinoma (RCC) accounts for more than 3% of all adult malignancies [1]. Tumor extension into the inferior vena cava (IVC), resulting in an IVC tumor thrombus (IVC‐TT), occurs in approximately 4%–10% of RCC cases [2]. RCC with IVC‐TT is usually associated with advanced disease and poor prognosis. Surgical resection remains the standard treatment; however, surgery for high‐level tumor thrombus is technically challenging and is associated with substantial perioperative morbidity and mortality [3].

Immune checkpoint inhibitors (ICIs) have transformed the management of advanced RCC. Programmed death‐ligand 1 (PD‐L1) expression has been investigated as a potential biomarker associated with ICI efficacy [4]. However, PD‐L1 expression alone has not been established as a definitive predictive biomarker, and the clinical role of ICIs in advanced RCC with IVC‐TT remains unclear.

Here, we report a case of advanced RCC with a Neves level 3 IVC‐TT [5] and interaortocaval lymph node enlargement. Pretreatment biopsy of the primary tumor demonstrated diffuse PD‐L1 expression, and nivolumab plus ipilimumab achieved a pathological complete response following surgery.

## 2 Case Presentation

**FIGURE 1:**
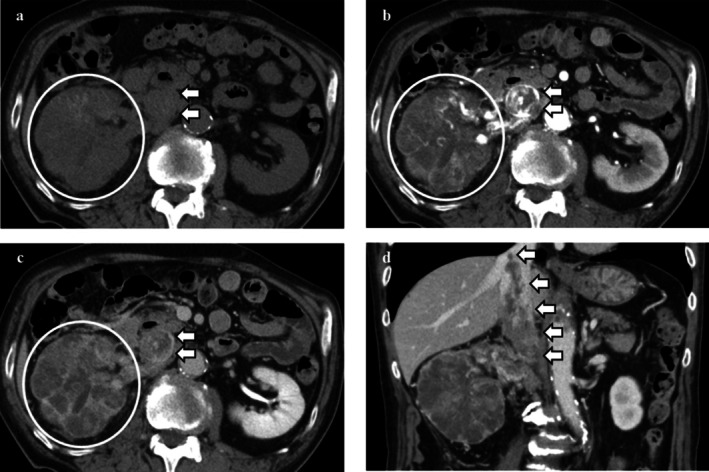
Dynamic abdominal CT at initial presentation. Dynamic abdominal CT at initial presentation revealed a large, heterogeneously enhancing renal mass measuring 91 mm in the right kidney. Focal areas of the mass showed strong enhancement in the corticomedullary phase with washout in the nephrographic phase, consistent with the typical contrast‐enhancement pattern of clear cell RCC. A tumor thrombus extending into the IVC up to the level of the hepatic veins was also observed. (a) Axial view: Non‐contrast phase; (b) Axial view: Corticomedullary phase; (c) Axial view: Nephrographic phase; (d) Coronal view: Corticomedullary phase. White circles indicate the renal mass, and white arrows indicate the tumor thrombus within the IVC.

**FIGURE 2:**
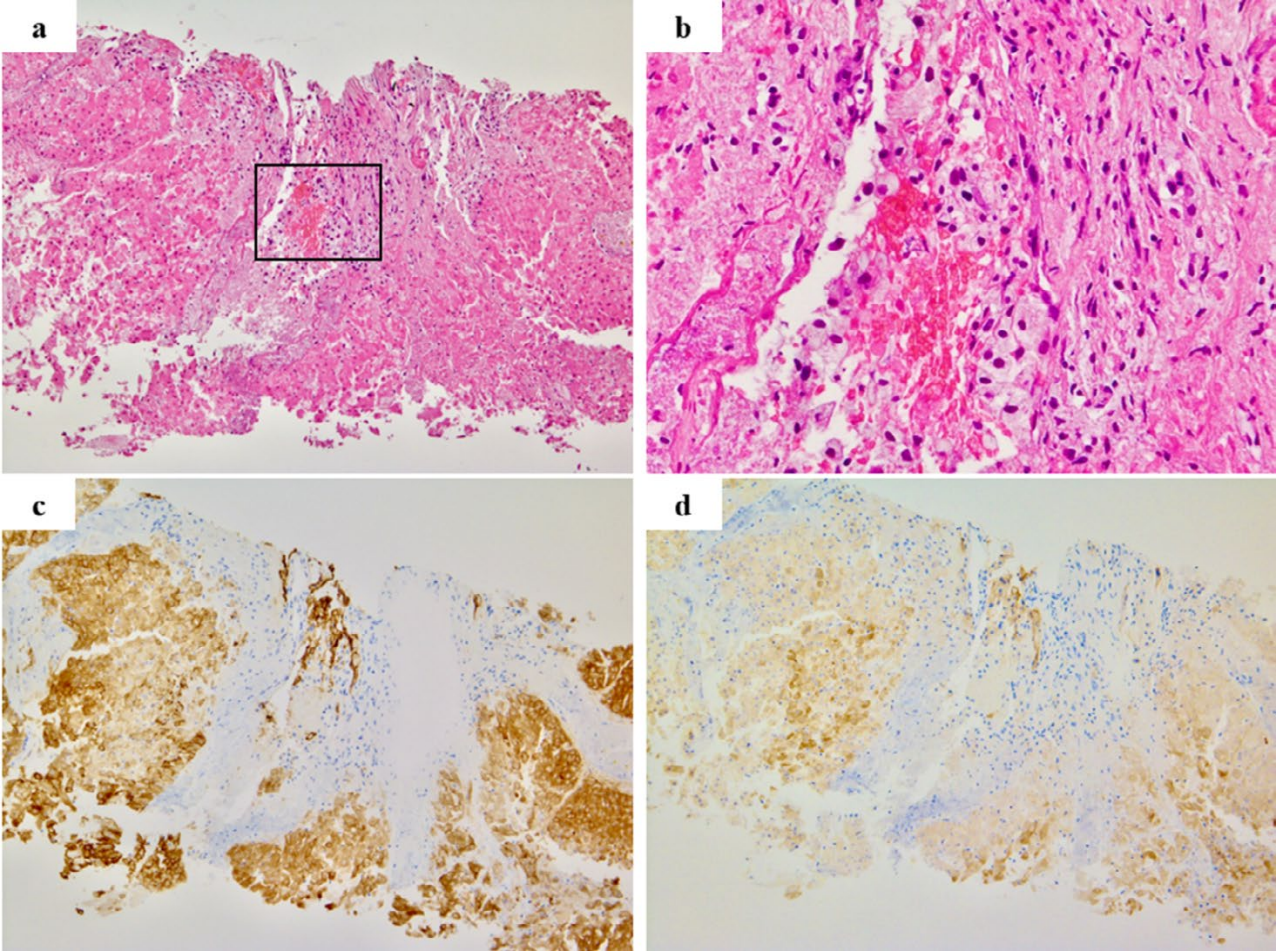
Hematoxylin and eosin and immunohistochemical staining of pretreatment CT‐guided needle biopsy specimens. (a) The biopsy specimen is predominantly composed of tumor cells with eosinophilic cytoplasm, interpreted as degenerative changes, arranged in an alveolar pattern (hematoxylin and eosin staining, ×40). (b) Higher‐magnification view of the boxed area in panel (a), showing a focal area with morphologic features consistent with clear cell RCC, without sarcomatoid/rhabdoid features, including tumor cells with clear cytoplasm (hematoxylin and eosin staining). (c) Immunohistochemical staining demonstrating diffuse positivity for carbonic anhydrase IX in tumor cells, supporting the diagnosis of clear cell renal cell carcinoma (×40; antibody: EP161, Cell Marque). (d) Programmed death‐ligand 1 immunohistochemical staining showing diffuse expression in nearly all tumor cells (tumor proportion and combined positive scores both approximately 100%) (×40; antibody: Ab228415, Abcam).

**FIGURE 3:**
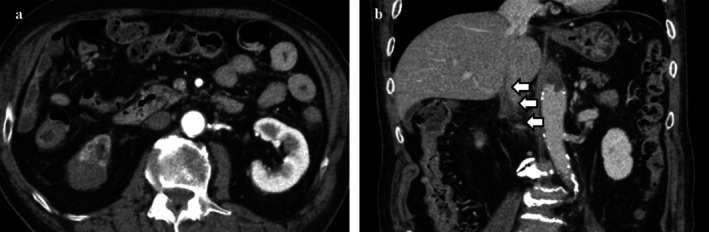
Preoperative contrast‐enhanced abdominal CT after 21 months of nivolumab plus ipilimumab therapy. Preoperative contrast‐enhanced CT performed 21 months after treatment initiation showed a reduction in renal tumor size to 29 mm (68.1% decrease from baseline) and regression of the IVC‐TT to 15 mm above the renal vein (Neves classification: Level 3 to level 1). (a) Axial view, corticomedullary phase; (b) Coronal view, corticomedullary phase. White arrows indicate the thrombus within the IVC.

**FIGURE 4:**
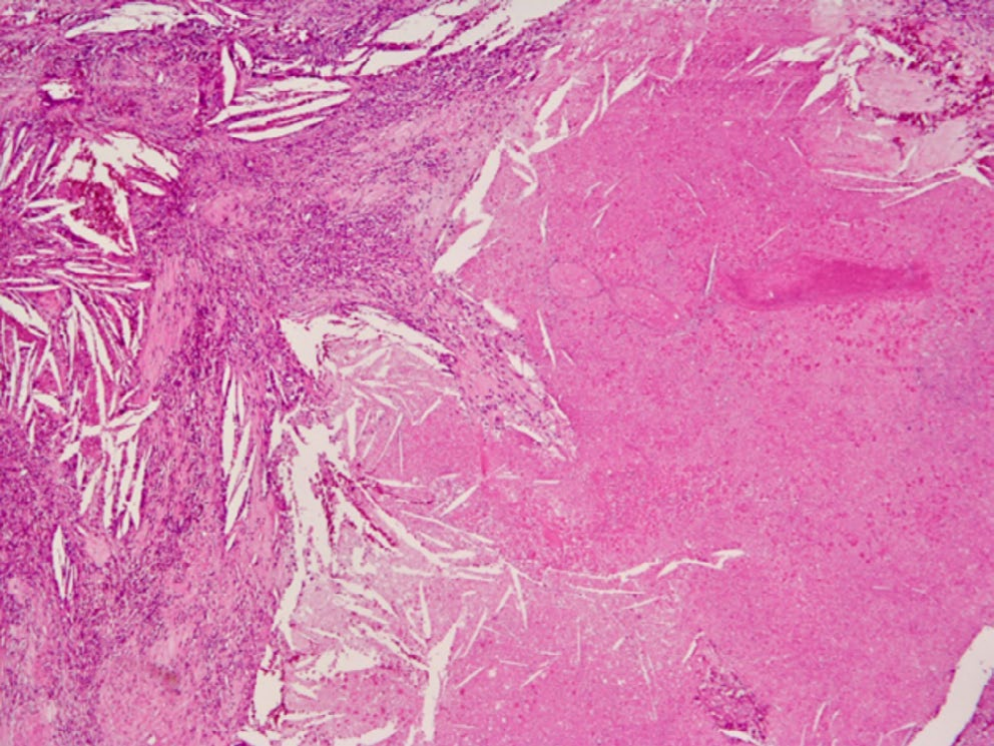
Hematoxylin and eosin staining of the resected tumor. Hematoxylin and eosin staining (×40) of the resected tumor showed extensive necrosis and inflammatory granulation tissue without viable tumor cells, indicating a pathological complete response (treatment effect grade 3).

A 78‐year‐old man underwent computed tomography (CT) for evaluation of abdominal pain at another hospital, which revealed a right renal mass with an IVC‐TT. He was referred to our department for further management. Contrast‐enhanced CT revealed a 91‐mm right renal mass with a typical enhancement pattern consistent with clear cell RCC, a tumor thrombus extending to the level of the hepatic veins, and a 15‐mm interaortocaval lymph node enlargement (Figure 1a–d). Laboratory findings included hemoglobin 10.9 g/dL, corrected calcium 10.3 mg/dL, albumin 2.8 g/dL, and C‐reactive protein 12.5 mg/dL. CT‐guided biopsy of the primary renal mass confirmed clear cell RCC without sarcomatoid/rhabdoid features, World Health Organization/International Society of Urological Pathology Grade 3 (Figure 2a–c). Immunohistochemical analysis revealed diffuse PD‐L1 expression in nearly all tumor cells (tumor proportion and combined positive scores both approximately 100%) (Figure 2d). The disease was staged as cT3bN1M0 and classified as poor‐risk according to the International Metastatic RCC Database Consortium criteria [6], with a Neves level 3 IVC‐TT [5]. The patient received nivolumab (240 mg) and ipilimumab (1 mg/kg) intravenously every 3 weeks for four cycles, followed by nivolumab 240 mg every 4 weeks. Two months after treatment initiation, C‐reactive protein levels normalized and hemoglobin levels improved, whereas serum calcium levels remained elevated until after surgery. Three months after treatment initiation, the patient developed Grade 3 adrenal insufficiency as an immune‐related adverse event, according to the Common Terminology Criteria for Adverse Events (version 5.0). This was managed with steroid replacement therapy, and nivolumab was continued. After 21 months of treatment, the renal mass decreased in size by 68.1%, IVC‐TT regressed from level 3 to level 1 (Figure 3a,b), and lymph node lesion resolved. Open right radical nephrectomy with IVC thrombectomy was subsequently performed in collaboration with general surgeons. The operative time was 7 h and 24 min, with an estimated blood loss of 3388 mL. The postoperative course was uneventful, and the patient was discharged 12 days after surgery. Histopathological examination showed extensive necrosis and inflammatory granulation tissue without viable tumor cells, indicating a pathological complete response; therefore, PD‐L1 expression in tumor cells could not be evaluated (Figure 4). On postoperative day 31, the patient developed a spontaneous intracerebral hemorrhage at home and subsequently died; postmortem examination confirmed no tumor‐related cause.

## 3 Discussion

We report a case of advanced RCC with high‐level IVC‐TT and lymph node metastasis that achieved a pathological CR following nivolumab plus ipilimumab therapy. Nearly all tumor cells in the pretreatment biopsy expressed PD‐L1, which may have been associated with the favorable response observed in this case.

RCC with IVC‐TT poses significant therapeutic challenges. Current guidelines recommend surgical resection [3], however, high‐level IVC‐TT is associated with perioperative mortality rates of approximately 10% and major complication rates of up to 34% [7]. Presurgical therapies have been explored to downstage IVC‐TT and reduce surgical morbidity [8]. To date, ICI combination therapies have been reported in a limited number of cases to be potentially effective as presurgical treatment for IVC‐TT [9, 10]. However, their clinical benefit has not yet been fully established. In the present case, 21 months of nivolumab plus ipilimumab resulted in marked regression of the IVC‐TT and complete resolution of nodal disease, enabling radical nephrectomy and IVC thrombectomy without thoracotomy. This clinical course suggests that presurgical ICI combination therapy followed by surgery may represent a valuable treatment strategy for selected patients with advanced RCC with IVC‐TT.

Nivolumab plus ipilimumab is an established first‐line therapy for intermediate‐ and poor‐risk advanced RCC, based on the phase III CheckMate 214 trial [4]. Although exploratory analyses demonstrated improved outcomes in patients with PD‐L1–positive tumors, subsequent biomarker analyses indicated that PD‐L1 expression alone is not a definitive predictive biomarker for response to this regimen [11]. However, network meta‐analyses further suggest that ICI‐TKI combinations generally achieve higher response rates in unselected populations with metastatic RCC, whereas nivolumab plus ipilimumab may confer a relative survival advantage in specific subgroups, including PD‐L1–positive disease [12]. Importantly, these analyses rely on population‐level data and binary PD‐L1 cut‐offs and do not account for patterns of PD‐L1 expression. Furthermore, several studies have reported that nivolumab plus ipilimumab induces weaker antitumor responses in primary tumors than in metastatic lesions [13]. Accordingly, deferred cytoreductive nephrectomy following initial disease control with ICI combination therapy has been proposed as a potential strategy to improve outcomes [14]. In the present case, pretreatment biopsy of the primary tumor demonstrated diffuse, tumor‐wide PD‐L1 expression (tumor proportion and combined positive scores both approximately 100%), a feature not captured by conventional PD‐L1 scoring systems. Although PD‐L1 expression alone is not an established predictive biomarker, this diffuse expression pattern may reflect a highly immune‐inflamed tumor microenvironment, potentially contributing to the profound pathological response observed in the primary tumor following nivolumab plus ipilimumab.

This case highlights two important implications. First, even in RCC with high‐level IVC‐TT, nivolumab plus ipilimumab can induce substantial tumor regression, thereby facilitating curative surgery. Second, although PD‐L1 expression alone is not a validated predictive biomarker, immunohistochemical features–such as diffuse PD‐L1 expression in pretreatment biopsy specimens–may be associated with favorable responses to nivolumab plus ipilimumab in individual cases. However, these findings should be interpreted with caution due to the intratumoral heterogeneity of RCC, particularly given that the pathological findings were obtained from biopsy specimens.

## Ethics Statement

The study protocol was approved by the Ethics Committee of Nippon Medical School Hospital (29‐11‐861) and was conducted in accordance with the Declaration of Helsinki.

## Consent

Written informed consent was obtained from the patient for the publication of this report.

## Conflicts of Interest

G.K. received lecture fees from Bristol‐Myers Squibb K.K. and Eisai Co. Ltd. All other authors declare no conflicts of interest.

## Data Availability

The datasets analyzed in the current study are available from the corresponding author upon reasonable request.
